# Gender Differences in Performance Predictions: Evidence from the Cognitive Reflection Test

**DOI:** 10.3389/fpsyg.2016.01680

**Published:** 2016-11-01

**Authors:** Patrick Ring, Levent Neyse, Tamas David-Barett, Ulrich Schmidt

**Affiliations:** ^1^Social and Behavioral Approaches to Global Problems, Kiel Institute for the World EconomyKiel, Germany; ^2^Medical Sciences Division, Department of Experimental Psychology, University of OxfordOxford, UK; ^3^Department of Economics, University of KielKiel, Germany; ^4^Department of Economics and Econometrics, University of JohannesburgJohannesburg, South Africa

**Keywords:** overconfidence, Cognitive Reflection Test, gender difference, performance prediction, competition, intra-gender competition

## Abstract

This paper studies performance predictions in the 7-item Cognitive Reflection Test (CRT) and whether they differ by gender. After participants completed the CRT, they predicted their own (i), the other participants’ (ii), men’s (iii), and women’s (iv) number of correct answers. In keeping with existing literature, men scored higher on the CRT than women and both men and women were too optimistic about their own performance. When we compare gender-specific predictions, we observe that men think they perform significantly better than other men and do so significantly more than women. The equality between women’s predictions about their own performance and their female peers cannot be rejected. Our findings contribute to the growing literature on the underpinnings of behavior in economics and in psychology by uncovering gender differences in confidence about one’s ability relative to same and opposite sex peers.

## Introduction

Confidence is an essential personality trait with a positive impact in numerous contexts, such as subjective well-being (Taylor and Brown, 1988, 1994), professional success (Kanter, 2004), or mental health (Taylor, 1989). *Over*confidence, on the other hand, is a psychological bias by definition, since it is an inaccurate judgment of one’s own abilities. Typical examples for this type of bias are overly optimistic beliefs in one’s professional abilities (Meyer et al., 2013) or physical fitness (Obling et al., 2015). This overly optimistic belief may be both absolute (i.e., individuals predict that their performance is better than it actually is) or relative (i.e., individuals predict that their performance is better than their peers’ when it actually is not). In the literature, the first type of overconfidence is referred to as overestimation and the latter as overplacement (Moore and Healy, 2008).

Overconfident beliefs appear to have positive consequences in some contexts, while they can be detrimental in others. Among other things, it has been suggested that overconfidence has positive psychological benefits, for example, on ambition, morale, and persistence (Pajares, 1996; Johnson and Fowler, 2011). Besides potential positive psychological benefits, overconfidence seems to help individuals in a social setting by convincing others that they have better skills or knowledge than they actually have (von Hippel and Trivers, 2011). Anderson et al. (2012) have shown empirically that individuals with high levels of overconfidence are perceived as more competent by their peers. This overstatement of one’s abilities could be an advantage in hiring decisions (Reuben et al., 2014).

Besides potential positive aspects, several empirical studies display the negative economic consequences of overconfidence. Camerer and Lovallo (1999), for example, have found that in a laboratory setting individuals tend to overestimate their chances of relative success and therefore excessively enter a competitive game. In a trading experiment, highly overconfident investors show less reaction to bad news, which results in lower profits for them compared to low overconfidence investors (Trinugroho and Sembel, 2011). Similarly, Barber and Odean (2001) have reported that overconfident investors reduce their net earnings by excessive trading; i.e., the expected gains from a trade do not exceed its transaction costs. Moreover, managerial overconfidence seems to explain investment distortion (Malmendier and Tate, 2005).

Despite the potential costs associated with overconfident beliefs in some settings, overconfident judgments are an integral part of various aspects of human decision making (De Bondt and Thaler, 1995). They are common in many professional fields such as investment banking (Stael von Holstein, 1972), economic negotiations (Neale and Bazerman, 1991), the law (Wagenaar and Keren, 1986), and even in clinical psychology (Oskamp, 1965). One typically observed pattern is that while both men and women are overconfident, men are more frequently prone to this bias than women (Lichtenstein et al., 1982; Lundeberg et al., 1994) and this seems to have important economic implications which will be discussed in the next subsection.

Barber and Odean (2001), for instance, have investigated the common stock investments of men and women separately. They have shown that men trade 45% more than women and this trading behavior actually reduces their earnings. They have concluded that this is likely due to greater overconfidence in men. Among other things, lower risk aversion in men can be attributed to higher overconfidence (Soll and Klayman, 2004). Furthermore, it has been shown in laboratory experiments that women are less likely to enter competition than men and lower levels of overconfidence are one explanation for this behavior (Niederle and Vesterlund, 2007; Reuben et al., 2012). It seems that women are disadvantaged in hiring decisions, because underconfident women may not appear as competent as their male peers (Reuben et al., 2014).

While the general tendency of men being more overconfident than women has been reported in several studies, less is known about the causes of this difference. This paper presents an experimental assessment of the extent to which this bias is driven by gender differences in confidence about one’s ability relative to same and opposite sex peers. Thereby, the paper extends the current literature (e.g., Dean and Ortoleva, 2015) on overconfidence by using gender-specific questions. This appears relevant from an economic perspective, as the composition of one’s potential competitors is important for individual decisions on whether to enter a competitive game (Datta Gupta et al., 2013). Beliefs about one’s self and the others have been shown to be important drivers for this decision (Camerer and Lovallo, 1999). Similar findings have been reported in evolutionary biology. In the course of human evolution, competition among men typically took place as direct and aggressive contests. Competition among women, by contrast, was typically more indirect and subtle (Stockley and Campbell, 2013). One potential explanation for these different types of behavior could be that the attractiveness of direct intra-gender competition is different for men and women, as they have different perceptions about their same-sex peers. Recent studies have applied evolutionary theory to explain decision-making patterns and this paper extends the literature to overconfidence. For example, it has been hypothesized that men, who face a higher sexual selection pressure than women (Trivers, 1972), should be more concerned about relative outcomes. Women, by contrast, should be more concerned about absolute outcomes, i.e., about resources for themselves and their offspring (Buss, 1989; Ermer et al., 2008). Following the predictions of this hypothesis, Schmidt et al. (2015) and Friedl et al. (2016) have shown that social comparison has a greater effect on men than on women in decision-making under risk and ambiguity.

In order to study gender differences in confidence about one’s ability relative to same and opposite sex peers, participants of this study first solved the 7-item Cognitive Reflection Test (CRT). Then they predicted their own (i), the other participants’ (ii), men’s (iii), and women’s (iv) number of correct answers in this task^1^. It was found that men perform better than women on this particular task, a result that has been previously reported (Kahneman and Frederick, 2002; Frederick, 2005). Moreover, it was observed that both men and women overestimate their performance; yet no significant gender effects in overestimation were found. When gender-specific predictions were compared, it emerged that men think they perform significantly better than other men. The equality between women’s predictions about their own performance and their female peers cannot be rejected.

## Materials and Methods

### Participants

Participants of the study were undergraduate students at Kiel University (*N* = 131; 72 women; mean age = 24.7). The experiment was organized and recruited with the software hroot (Bock et al., 2014). The participants were randomly seated in a classroom in groups of 15. They first read the general instructions for the experiment themselves; then the instructions were read out loud. After the protocol was completed, they were invited to a separate room to get paid anonymously. The protocol also included a short questionnaire on life satisfaction questions and digit ratio measurement. Evidence obtained on the relation between overconfidence scores and digit ratios from this experiment is reported in Neyse et al. (2016).

### 7-item CRT

The 7-item CRT (Toplak et al., 2014) is an extended version of the original 3-item CRT (Frederick, 2005) that includes four additional questions. The CRT is designed to observe participants’ ability to activate the Type 2 cognitive process instead of giving intuitive and effortless answers through the Type 1 cognitive process. According to the dual process theories of cognition (Kahneman and Frederick, 2002), the Type 1 cognitive process yields to intuitive and automatic reasoning, while the Type 2 process requires more thorough thinking and conscientiousness. The first question of the CRT is as follows:

“*A bat and a ball together cost $1.10. A bat costs $1 more than a ball. How much does a ball cost?*”

The intuitive, but incorrect, answer is 10 cents. The correct answer is 5 cents.

### Performance Predictions

Participants first received the 7-item CRT, which they had to complete within 10 min. After 10 min, the answer sheets were collected. This way, participants were prevented from making any changes on the answer sheets, since their predictions were also incentivized. Following the CRT, they were given another sheet on which they were asked to predict their own number of correct answers (i), the average number of correct answers of other participants in their group (ii), the men in their group (iii), and the women in their group (iv). For each correct answer in the CRT, the participants were paid €0.5. Correct predictions about their own score and others’ scores were rewarded with €2 and false predictions with nothing. Gender-specific predictions were not incentivized^2^. The prediction task was not announced beforehand in order to avoid strategic behavior in answering the 7-item CRT itself. Participants used pen and paper to answer both the CRT-questions and the prediction task. Instructions for the experiment can be found in the Supplementary Material.

### Ethics Statement

All participants of the experiment were informed about the content and the protocol of the study before participation. Their anonymity was preserved by assigning them a randomly generated code that cannot be associated with any personal information or decision. As is standard in economics experiments, no ethical concerns were involved other than preserving the anonymity of the participants. The whole protocol was performed in accordance with the Declaration of Helsinki and conformed to the ethical guidelines of the Kiel University Experimental Economics Lab, where it was approved by the lab manager.

## Results

### Summary Statistics

**FIGURE 1:**
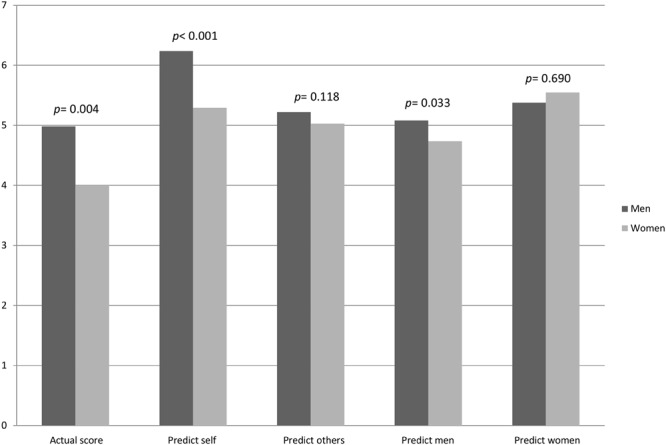
**Average scores and predictions by gender**.

**Figure 1** presents the means of actual scores and predictions by gender. The participants scored 4.44 (*SD* = 1.836) correct answers on average regardless of gender^3^. Mean number of correct answers for men is 4.98 (*SD* = 1.892) and for women 4.00 (*SD* = 1.914). A two-sample Wilcoxon rank-sum test confirms that the average score of men is significantly higher than of women (*z* = -2.847, *p* = 0.004). This is in line with previous findings in the literature (Kahneman and Frederick, 2002; Frederick, 2005; Cueva et al., 2016).

Participants predicted that they themselves had answered 5.72 (*SD* = 1.416) questions correctly on average. Men predicted their own scores as 6.24 (*SD* = 1.165), while women predicted their own scores as 5.29 (*SD* = 0.173). A Wilcoxon rank-sum test confirms that this gender difference is significant (*z* = -4.144, *p* < 0.001).

The overall predictions about the other participants’ number of correct answers is 5.12 (*SD* = 0.966) on average. Men’s mean prediction is 5.22 (*SD* = 1.115), while women’s is 5.03 (*SD* = 0.822). The two-sample Wilcoxon rank-sum test does not reject the null hypothesis of no difference (*z* = -1.564, *p* = 0.118).

In addition to predictions about their own and other participants’ performance, participants were also asked to predict the average scores of men and women in their group separately. The prediction about men’s mean scores is 4.89 (*SD* = 1.109) and about women’s is 5.47 (*SD* = 0.998) for the whole sample. Men’s prediction about other men is 5.08 (*SD* = 1.204) and women’s prediction about men is 4.74 (*SD* = 1.007). The difference is statistically significant (*z* = -2.129, *p* = 0.033). Men predicted women’s score as 5.38 (*SD* = 1.117) and women’s average prediction about women was 5.55 (*SD* = 0.885). Non-parametric analysis does not confirm a statistically significant difference (*z* = 0.400, *p* = 0.690)^4^.

**Table 1** presents the comparison analysis of predictions. All results are gathered with Wilcoxon signed-rank tests. *p*-values are given for all participants as well as for men and women separately. Inequality signs to the right of each *p*-value indicate whether the value of the difference between the two predictions in the first column is positive, zero, or negative. Differences in means are not reported in **Table 1** as they are available in **Figure 1**. The first row shows that both men and women overestimate their scores. Their predictions about their own scores are significantly higher than their actual scores (*p* < 0.001). This result is a clear indication of overestimation, which is the difference between one’s actual score and prediction. The second row shows that both men and women predicted that they would do better than other participants (*p* < 0.001 for men and *p* = 0.050 for women). This result is an indication of overplacement. Gender-specific predictions indicate that both men and women thought they did better than men (*p* < 0.001 for both). Yet, only men thought they did better than women (*p* < 0.001 for men and *p* = 0.104 for women). Finally gender-specific predictions are compared with each other. Row 5 shows that both men and women thought women would do better than men on the task (*p* < 0.001 for women and *p* = 0.004 for men).

**Table 1:** Comparisons of actual performance and predictions about others.

	**All**		**Men**		**Women**	
Self vs actual score	*p* < 0.001	>	*p* < 0.001	>	*p* < 0.001	>

**Comparisons of predictions**						
Self vs others	*p* < 0.001	>	*p* < 0.001	>	*p* = 0.050	>
Self vs men	*p* < 0.001	>	*p* < 0.001	>	*p* < 0.001	>
Self vs women	*p* = 0.023	>	*p* < 0.001	>	*p* = 0.104	–
Men vs women	*p* < 0.001	<	*p* = 0.004	<	*p* < 0.001	<

*Self, *others, men*, and *women* denote predictions whereas *actual score* does not.*

### Gender-Specific Differences in Performance Predictions

The main research questions are whether there are gender-specific differences in overestimation and overplacement scores and whether such gender-specific differences can be related to participants’ gender biases about other participants’ performance. In order to answer them, four different variables based on participants’ predictions and their actual performance were generated (**Table 2**).

**Table 2:** Generated overestimation and overplacement variables.

				Min	Max
Overestimation	Actual score	–	Prediction about one’s performance	-2	6
Overplacement	Prediction about one’s performance	–	Prediction about the others’ performance (all)	-2	5
Intra-gender overplacement	Prediction about one’s performance	–	Prediction about the others’ performance (own gender)	-3	5
Inter-gender overplacement	Prediction about one’s performance	–	Prediction about the others’ performance (other gender)	-2	5

*Min (max) is the smallest (largest) value of the respective variable in our data-set.*

Overestimation is the difference between one’s self-prediction and actual score, and overplacement is the difference between one’s self-prediction and the prediction about others regardless of gender. According to Moore and Healy (2008) overestimation and overplacement are two aspects of overconfidence^5^. The intra-gender overplacement variable detects how much better or worse one thinks she/he is than the other participants with the same gender. Likewise, the inter-gender overplacement variable shows how much better or worse one thinks she/he is than participants of the other gender.

**FIGURE 2:**
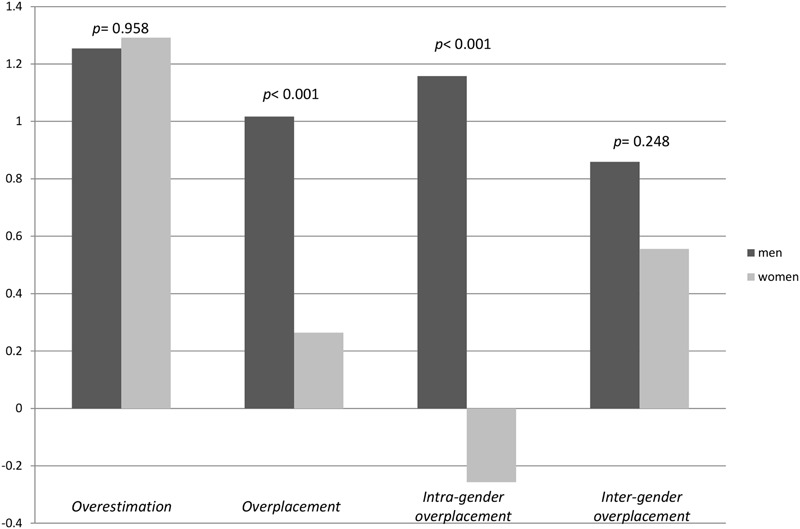
**Average overestimation and overplacement scores**.

Both men and women in our sample overestimated their own scores (**Figure 2**). The average overestimation score for men is 1.25 (*SD* = 1.409) and for women 1.29 (*SD* = 1.542). A Wilcoxon rank-sum test does not detect any statistically significant gender difference in overestimation scores (*z* = 0.053, *p* = 0.958). Yet, men tend to overplace themselves significantly more than women (*z* = -3.737, *p* < 0.001). The average overplacement score is 1.02 (*SD* = 1.025) for men and 0.26 (*SD* = 1.138) for women. Intra-gender overplacement is significantly higher in men than women (*z* = -5.942, *p* < 0.001). Men’s average intra-gender overplacement score is 1.16 (*SD* = 1.142) and women’s is -0.26 (*SD* = 1.303). However, significant gender differences in inter-gender overplacement were not observed (*z* = -1.155, *p* = 0.248). The inter-gender overplacement scores are 0.86 (*SD* = 1.058) for men and 0.56 (*SD* = 1.047) for women.

In a nutshell, we observe that men think they perform significantly better than other men and do so significantly more than women. The equality between women’s predictions about their own performance and their female peers, however, cannot be rejected.

## Discussion

The main outcome of the study is that men think that they would perform significantly better on the 7-item CRT than their male peers, while women made comparable predictions about their own performance and their female peers. This gender-specific overplacement variable is significantly different between men and women with men overplacing their performance more than women.

A large body of literature in economics and psychology suggests that women, on average, are less confident and competitive than men (see Croson and Gneezy, 2009 for an overview). We contribute to this literature by uncovering gender differences in confidence about one’s performance relative to same and opposite sex peers. Previous research has indicated that social components in a choice situation have an impact on gender differences in confidence and competitive behavior. On the one hand, it has been shown that women are more confident in their group’s performance than in their own performance, while men are less confident in their group’s performance compared to their own (Healy and Pate, 2007). While this study indicates that predictions about one’s own and other’s performance might differ by gender in certain situations, it does not specifically assess whether differences in performance are due to gender distribution within the reference group. Therefore, it is not directly comparable to the present study, where each participant was specifically asked about her/his prediction about men’s and women’s performance separately. On the other hand, it has been shown that men’s decision to enter a tournament or a piece-rate pay scheme can depend on the co-participant’s gender (Datta Gupta et al., 2013). In that study, men competed less against other men than against women, when the gender information was made sufficiently salient. While this result appears to be out of line with our findings, which might be due to the task type or the transition from beliefs to actions, it shows that competitive behavior might have intra- and inter-gender-specific components. This is a finding that is also often reported in the context of evolutionary-biology, which we refer to in the next part of the discussion.

In the course of human evolution, competition among males typically took place as direct and aggressive contests. Competition among females, by contrast, often occurred more indirectly and subtly. One potential explanation for these different types of behavior could be that the attractiveness of direct intra-gender competition is different for men and women, as they have different perceptions about their same-sex peers. It may be suggested that confidence in one’s own abilities relative to one’s competitors is an important drive underlying this observation. The link between beliefs about relative skill and the decision to enter competition has been established by several economics experiments (Camerer and Lovallo, 1999). If men think that they perform better than their peers (other men), it potentially makes direct competitions attractive for them. If women, by contrast, think that their peers (other women) perform similarly to them, direct competition appears less attractive and competition might take place on a more subtle level.

It appears important, however, to stress the possibility of reversed causality. It might be that evolutionary differences in competitiveness as an attitude may affect confidence beliefs due to self-enhancement. Self-enhancement refers to the fact that individuals gain positive utility by comparing themselves with lower ranked peers (Wood and Taylor, 1991). In particular, due to evolutionary differences between the levels of male and female competitiveness, confidence beliefs may give them different utility levels. Falk and Knell (2004) developed a social comparison model that includes self-enhancement and self-improvement in the utility function. The model shows that people with higher abilities tend to compare themselves with people who also have high abilities. They also show that women have lower reference standards. This finding is in line with our results showing that women over-place themselves less than men.

Some words of caution are in order. First, this study is on predictions about others relative to one’s performance. In future studies whether the above outlined causal chain from beliefs about performance translates into actual competitive behavior should be addressed. Second, performance predictions for the CRT were studied. This is a special task that aims on impulsiveness of decision-making and to what extent our findings apply in a broader context deserves further investigation.^6^ Dreber et al. (2014), for example, have shown that the type of task matters with respect to gender differences in competitive behavior. Third, confidence and competition are social notions that develop via countless interactions in distinct contexts. Due to its specific research questions, the design of the current study does not involve any social interaction between participants. Yet, it may be the case that overconfident behavior can alter with human interaction or social motives. For example, Burks et al. (2013) have shown that overconfidence can be induced by the desire to send positive signals to others about one’s own skill. Therefore, attention needs to be paid to the role of social interaction and motives on overconfidence in future studies.

## Author Contributions

All authors listed, have made substantial, direct and intellectual contribution to the work, and approved it for publication.

## Conflict of Interest Statement

The authors declare that the research was conducted in the absence of any commercial or financial relationships that could be construed as a potential conflict of interest.

## Footnotes

1 Note that we use the word *predict* to refer to participants’ guesses throughout the article, which should not be confused with the term *prediction* in econometrics.

2 Since the gender information was gathered in a different sheet of paper at the end of the protocol and due to time constraints, we did not incentivize the gender-specific predictions. A two-sample variance comparison test did not reject the null hypothesis that the prediction variance is equal for unincentivized and incentivized predictions for both men and women at the 5% significance level.

3 If we consider only the first three items as in Frederick (2005), the average number of correct answers is 1.76 (*SD* = 1.068). In contrast to previous studies (Brañas–Garza et al., 2015; Cueva et al., 2016), the number of participants who answered none of the questions correctly was rather low in our experiment. See Supplementary Figures A1 and A2 for histograms on the distribution of correct answers.

4 Since there was no interaction between participants and since the performance of participants was not disclosed, group behavior cannot affect the individual behavior. Therefore, no possible reflection problem is anticipated (Manski, 1993).

5 The third aspect is excessive precision, yet we only focus on the first two in the current study.

6 See Cueva et al. (2016) for a review of reflective and non-reflective responses in CRT.
