# Supplementary material for: Gender Differences in Performance Predictions: Evidence from the Cognitive Reflection Test
Source: Front Psychol. 2016 Nov 1;7:1680. doi: 10.3389/fpsyg.2016.01680 (PMC5089055; doi:10.3389/fpsyg.2016.01680)

## Appendix

### A1. Instructions for the 7-item CRT

Please answer the following questions. For each correct answer you will earn € 0.5.

Attention: They are mathematical questions which can be either right or wrong. You have ten minutes to answer all questions.

For the specific questions, please refer to Toplak et al. (2014).

### A2. Instruction for the predictions

In the following we would like to receive your answers to the following questions. For each correct answer you will earn € 2 (for question 1 and 2 only). The results will be rounded to the next integer.

1. How many questions did you answer correctly?
2. How many questions did the other participants, on average, answer correctly?
3. How many questions did the female participants, on average, answer correctly?
4. How many questions did the male participants, on average, answer correctly?

**Figure A1 Distribution of correct answers in 3-item CRT**

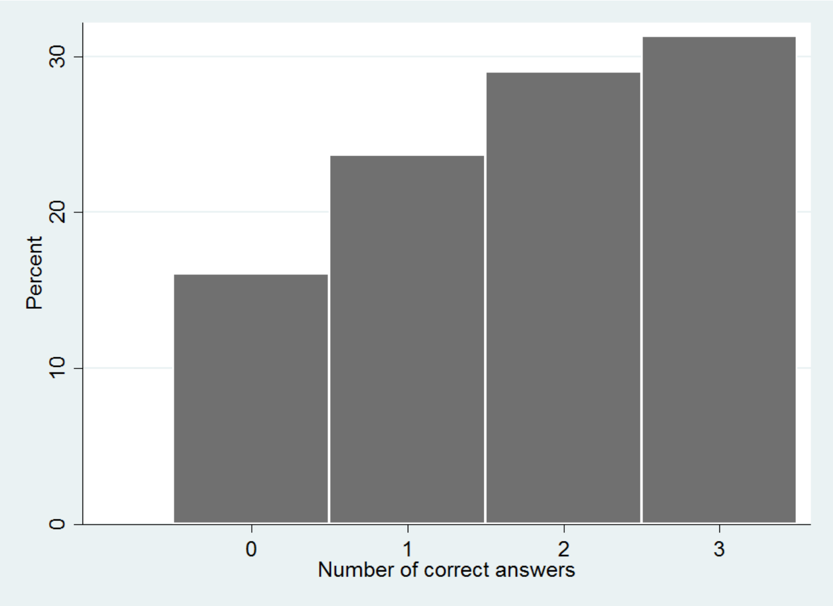


**Figure A2 Distribution of correct answers in 7-item CRT**

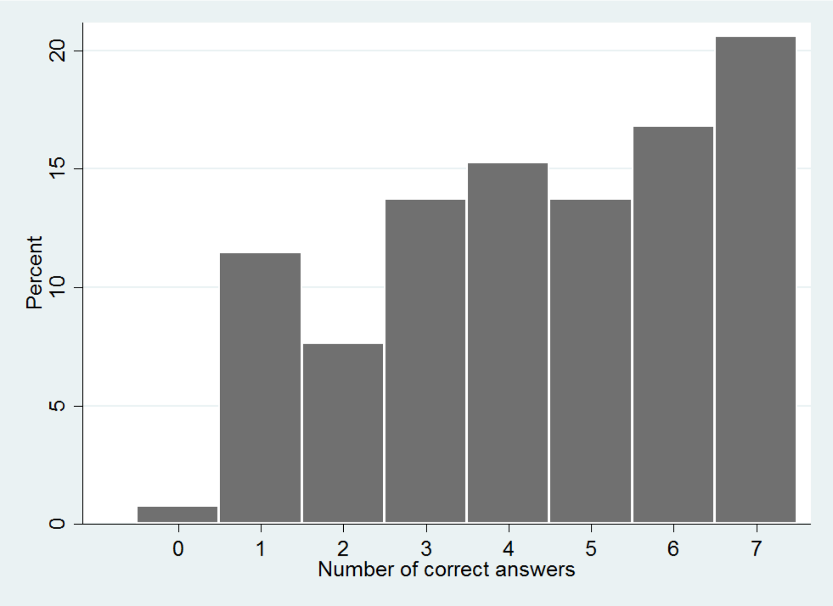

Supplement: Supplementary file 1 [file Data_Sheet_1.PDF]
